# The evaluation of kidney function estimation during lifestyle intervention in children with overweight and obesity

**DOI:** 10.1007/s00467-024-06435-0

**Published:** 2024-07-04

**Authors:** Mark J. C. M. van Dam, Hans Pottel, Pierre Delanaye, Anita C. E. Vreugdenhil

**Affiliations:** 1 Centre for Overweight Adolescent and Children’s Healthcare (COACH), Department of Pediatrics, School of Nutrition and Translational Research in Metabolism (NUTRIM), Maastricht University Medical Center +, MosaKids Children’s Hospital, P. Debyelaan 25, 6229 HX Maastricht, The Netherlands; 2 Department of Public Health and Primary Care, KU Leuven Campus Kulak Kortrijk, Kortrijk, Belgium; 3 Department of Nephrology-Dialysis-Apheresis, Hôpital Universitaire Carémeau, Nîmes, France; 4 Department of Nephrology-Dialysis-Transplantation, University of Liège, CHU Sart Tilman, Liège, Belgium

**Keywords:** Childhood obesity, Creatinine, eGFR, Pediatrics, Lifestyle intervention

## Abstract

**Background:**

Children with overweight and obesity are at risk for developing chronic kidney disease (CKD). During lifestyle adjustment, the first step in the treatment of childhood obesity, body proportions are likely to change. The aim of this study was to examine how lifestyle intervention affects creatinine-based kidney function estimation in children with overweight and obesity.

**Methods:**

This longitudinal lifestyle intervention study included 614 children with overweight and obesity (mean age 12.17 ± 3.28 years, 53.6% female, mean BMI *z*-score 3.32 ± 0.75). Loss to follow-up was present: 305, 146, 70, 26, and 10 children were included after 1, 2, 3, 4, and 5 (about yearly) follow-up visits, respectively. Serum creatinine (SCr) was rescaled using *Q*-age and *Q*-height polynomials.

**Results:**

At baseline, 95–97% of the children had a SCr/Q-height and SCr/Q-age in the normal reference range [0.67–1.33]. SCr/Q significantly increased each (about yearly) follow-up visit, and linear mixed regression analyses demonstrated slopes between 0.01 and 0.04 (corresponding with eGFR FAS reduction of 1.1–4.1 mL/min/1.73 m^2^) per visit. BMI *z*-score reduced in both sexes and this reduction was significantly higher in males. No correlation between change in rescaled SCr and BMI *z*-score reduction could be demonstrated.

**Conclusions:**

Rescaled serum creatinine (SCr/Q) slightly increases during multidiscipline lifestyle intervention in this cohort of children with overweight and obesity. This effect seems to be independent from change in BMI *z*-score. Whether this minor decrease in estimated kidney function has clinical consequences in the long term remains to be seen in trials with a longer follow-up period.

**Clinical Trial registration:**

ClinicalTrial.gov; Registration Number: NCT02091544.

**Graphical abstract:**

**Figure:**
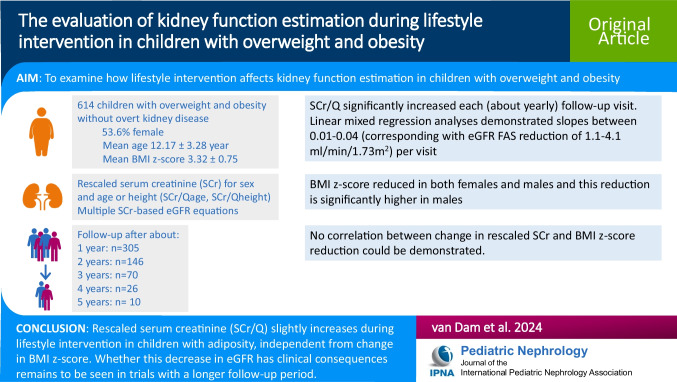
A higher resolution version of the Graphical abstract is available as Supplementary information

**Supplementary Information:**

The online version contains supplementary material available at 10.1007/s00467-024-06435-0.

## Introduction

In the last few decades, it has become widely accepted that obesity is harmful for the kidneys [1]. Obesity is known to aggravate primary kidney diseases, both congenital and acquired [2–4]. In addition, obesity is an independent risk factor for chronic kidney disease (CKD) in patients with no underlying kidney disease [5] and obesity-related glomerulopathy is a distinct proteinuric kidney disease with its own histological characteristics [6, 7]. A hallmark of obesity-related kidney disease is glomerular hyperfiltration, a state of increased glomerular filtration rate (GFR). This hyperfiltration can be “absolute,” in cases of a normal number of functioning nephrons, or “relative,” in cases of congenital or acquired reduced number of functioning nephrons [8]. A high GFR is often used as a surrogate marker for hyperfiltration, a controversial topic since it is still impossible to exactly determine nephron mass in a clinical setting [9].

Serum creatinine (SCr) is the endogenous marker of first choice to estimate kidney function. Although its properties make it an almost ideal marker, multiple factors can influence SCr apart from kidney function, such as muscle mass [10]. SCr during childhood is heavily age-dependent because levels of SCr increase with muscle mass and thus with age and height. Between the age of 2 and 14 years, SCr increases in a linear way with no differences between sexes. Thereafter, on average, SCr increases in females to a level of 0.7 mg/dL and in males to 0.9 mg/dL at the age of 18 years [11].

Most children with overweight and obesity without known kidney disease have a SCr within the normal reference range for children [12] and it is known that the choice of SCr-based GFR-estimating equation has an enormous impact on the value of estimated GFR (eGFR) [13]. During a lifestyle intervention program, especially when this includes increased physical activity, body proportions (e.g., fat and muscle mass ratio) are likely to change and it is therefore plausible that SCr will change as well. Since increased physical activity leads to more muscle mass, SCr might increase as a result.

Lifestyle adjustment is the first step in the treatment of obesity in both children and adults, and data on how lifestyle intervention affects serum creatinine and thus kidney function estimation in children are lacking. Therefore, the goals of this study are the following:

- to evaluate the course of SCr levels during a lifestyle intervention in children with overweight and obesity;
- to examine what happens with rescaled SCr for sex and age or height (SCr/Q-age and SCr/Q-height, respectively);
- to examine whether all these findings are different in children stratified according to BMI *z*-score response during the lifestyle intervention program.

## Methods

### Setting and study inclusion

**Fig. 1:**
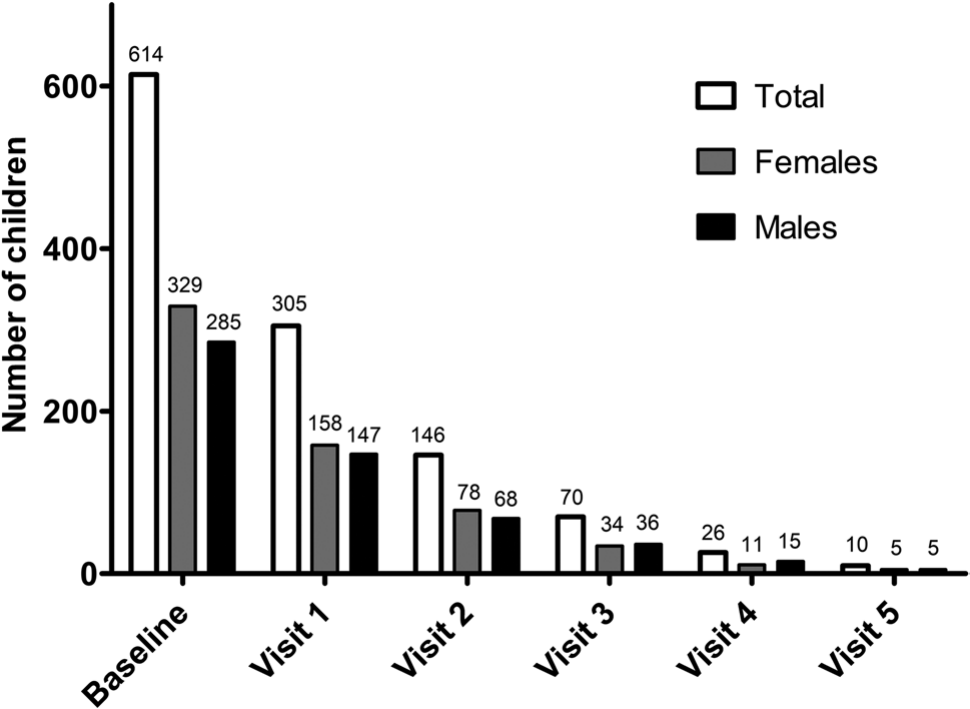
Number of children at baseline and during the follow-up study, displayed for both females and males

For this study, both baseline and follow-up data were used from children participating in the Centre for Overweight Adolescent and Children’s Healthcare (COACH) at Maastricht University Medical Centre + (MUMC +), MosaKids Children’s Hospital. COACH offers children and their families a personalized multidisciplinary lifestyle intervention with monthly evaluations in the outpatient department of pediatricians, physiotherapists, dieticians, and social workers among others, as described previously [14]. Both at baseline and about yearly (not necessarily every 12 months, median 14.6 months (interquartile range 12.7–17.5 months) in between baseline and first follow-up moment), children are evaluated both clinically and by means of laboratory tests among others. Between January 1, 2011, and April 1, 2019, 662 children entered the program. Excluded for this study were children with secondary causes of overweight, congenital or acquired kidney disease, diabetes mellitus, and/or current use of antihypertensive medication (*n* = 13). Twenty-five children were excluded because of missing data. Five participants were older than 18 years at clinical evaluation and 5 children did not meet the criteria for overweight or obesity. Finally, 614 children were included in the baseline study. Loss to follow-up was present and not all children who continued the lifestyle intervention program agreed with yearly blood analyses and were therefore considered lost to follow-up for this particular study. Therefore in this longitudinal study, 305, 146, 70, 26, and 10 children were included after 1, 2, 3, 4, and 5 years, respectively, presented in Fig. 1. This study was approved by the Medical Ethical Committee of the MUMC + and met the guidelines administered by the Declaration of Helsinki. Informed consent was obtained from all children 12 years or older, and from all parents or legal guardians.

### Clinical assessment and anthropometry

Both at baseline and about yearly, children underwent physical examination and measurement of weight and height (using a digital scale (Seca, Chino, CA) and digital stadiometer (De Grood Metaaltechniek, Nijmegen, the Netherlands), respectively). Overweight, obesity, and severe obesity were defined according to the International Obesity Task Force (IOTF) criteria [15]. Clinically relevant BMI *z*-score reduction was defined as a changed BMI *z*-score (BMI *z*-score baseline minus BMI *z*-score of last visit) of 0.25 and 0.50 [16]. The equation by Haycock et al. was used to calculate body surface area (BSA) out of height (cm) and weight (kg), $BSA=0.024265 \times {Wt}^{0.5378}\times {Ht}^{0.3964}$) [17]. In a subgroup of randomly selected children (*n* = 291, 88, 50, 25, 7, and 0 at baseline and follow-up moment 1, 2, 3, 4, and 5, respectively; data was insufficient for paired analyses), fat and fat-free masses were determined using air displacement plethysmography (BodPod).

### Serum creatinine (SCr), rescaling of SCr for sex and age or height, and SCr-based eGFR

The enzymatic method (Cobas 8000, Roche) was used to determine serum creatinine (SCr). Since SCr during childhood is heavily dependent on age and height (and sex from the age of 14 years), SCr was normalized using references values obtained from the literature [11, 18], resulting in *Q*-age and *Q*-height, in which *Q* is the median SCr for healthy children according to sex and age (*Q*-age) [19] or height (*Q*-height) [18]. SCr/*Q* is expected to be “1” for healthy children, and values of 0.67 and 1.33 represent the 2.5th and 97.5th percentiles of the normal reference range [20]. SCr was converted into eGFR using the following equations: full-age spectrum (FAS), both height independent (FAS-age) and height dependent (FAS-height) [18, 19]; new European Kidney Function Consortium (EKFC) [21]; updated bedside Schwartz or CKiD [22]; Schwartz-Lyon [23]; CKiD under 25 years (CKiDU25) [24]; adjusted-creatinine revised Lund-Malmö (LMR18) [25]; and CKD-EPI equation with age-adjusted creatinine values (CKD-EPI40) [26].

### Statistical analysis

Summary statistics are presented as mean ± standard deviation (for normally distributed data) and median (interquartile range) otherwise. Delta of paired variables was calculated out of value of the last visit minus baseline value. Comparisons between groups were made with the independent samples *t*-test for normally distributed data and the independent samples Mann–Whitney *U* test otherwise. All *P*-values are two tailed and a *P*-value below 0.05 was considered statistically significant. Statistics were performed with SAS 9.4 (SAS Institute Inc., Cary, NC, USA).

## Results

### Baseline

At baseline, 614 children were included of whom 329 were female (53.6%) and 285 were male (46.4%), demonstrated in Table 1. Mean age was 12.17 ± 3.28 years and mean BMI *z*-score was 3.32 ± 0.75. Overweight, obesity, and severe obesity was present in 128 (20.8%), 274 (44.6%), and 212 (34.5%) of the children, respectively. Mean percentage of fat mass of total body weight was 44.15 ± 6.37 percent (*n* = 291). Mean SCr/*Q*-age was 0.99 ± 0.16 and SCr/*Q*-height was 0.96 ± 0.17. Of the children, 593 (96.6%) and 582 (94.8%) had a SCr/*Q*-age and SCr/*Q*-height in the normal reference range of 0.67–1.33. Mean eGFR was between 95.4–113.4 mL/min/1.73 m^2^ and 101.9–118.6 mL/min/1.73 m^2^ for females and males, respectively, depending on the eGFR equation used.

**Table 1:** Baseline characteristics of the children with overweight and obesity stratified according to sex

Variable	Total cohort (*n* = 614)	Female (*n* = 329)	Male (*n* = 285)	*P*-value
Age (years)	12.17 ± 3.28	12.41 ± 3.43	11.90 ± 3.09	0.027*
Weight (kg)	73.1 ± 27.7	73.2 ± 27.6	73.1 ± 27.8	0.481
Height (cm)	154.0 ± 17.0	153.1 ± 16.3	155.0 ± 17.7	0.091
BSA (m^2^)	1.78 ± 0.43	1.77 ± 0.43	1.78 ± 0.43	0.398
BMI *z*-score	3.32 ± 0.75	3.14 ± 0.70	3.52 ± 0.76	< 0.001*
Fat mass (% of total body weight) ^1^	44.15 ± 6.37	43.69 ± 6.35	44.62 ± 6.38	0.107
SCr (mg/dL)	0.58 ± 0.14	0.58 ± 0.13	0.58 ± 0.14	0.271
SCr/*Q*-age	0.99 ± 0.16	1.01 ± 0.15	0.97 ± 0.15	< 0.001*
SCr/*Q*-height	0.96 ± 0.17	0.98 ± 0.17	0.93 ± 0.15	< 0.001*
eGFR FAS-age	111.0 ± 18.1	108.6 ± 17.0	113.8 ± 19.4	< 0.001*
eGFR FAS-height	115.8 ± 20.6	113.4 ± 20.9	118.6 ± 20.2	< 0.001*
eGFR EKFC	103.9 ± 11.1	101.4 ± 11.7	106.8 ± 10.5	< 0.001*
eGFR CKiD (‘bedside Schwartz’)	113.8 ± 20.0	112.5 ± 20.3	115.3 ± 19.6	0.043*
eGFR Schwartz-Lyon	103.5 ± 17.6	100.2 ± 18.1	107.3 ± 17.0	< 0.001*
eGFR CKiD-U25	106.6 ± 17.0	100.8 ± 16.4	113.2 ± 17.8	< 0.001*
eGFR LMR18	98.4 ± 10.9	95.4 ± 10.8	101.9 ± 11.0	< 0.001*
eGFR CKD-EPI40	100.4 ± 13.2	96.6 ± 13.8	104.7 ± 12.5	< 0.001*

Data presented as mean ± standard deviation. ^1^ Data was available for 291 children (147 females, 144 males). *P*-value is obtained with the independent samples *t*-test. A *P*-value below 0.05 was considered statistically significant

*BSA* body surface area, *BMI* body mass index, *SCr* serum creatinine, *SCr/Q-age* serum creatinine normalized using Q-age polynomials, *SCr/Q-height* serum creatinine normalized using Q-height polynomials, *FAS* full-age spectrum, *EKFC* European Kidney Function Consortium, *CKiD* chronic kidney disease in children, *CKiDU25* CKiD under 25, *LMR18* revised Lund-Malmö extended to children, *CKD-EPI40* Chronic Kidney Disease Epidemiology Collaboration extended to children

Compared to females, males were slightly younger (12.41 years vs. 11.90 years, *p* = 0.027), had a higher BMI *z*-score (3.14 vs. 3.52, *p* < 0.001), and significantly lower SCr/Q and higher eGFR.

### Follow-up

Loss to follow-up was present and not all children who continued in the lifestyle intervention program agreed to yearly blood analyses. Therefore, in the longitudinal study, 305, 146, 70, 26, and 10 children were included after 1, 2, 3, 4, and 5 repeated measurements, respectively, as presented in Fig. 1. Compared to children who were lost to follow-up after baseline measurement (*n* = 309), children with ≥ 1 follow-up measurement(s) (*n* = 305) were significantly younger at baseline (11.68 ± 3.00 years vs. 12.66 ± 3.47 years, *p* < 0.001), but did not differ concerning sex, BMI *z*-score, SCr/*Q*-age, and SCr/*Q*-height. Compared to children with exactly one follow-up measurement (*n* = 159), children with ≥ 2 follow-up measurements (*n* = 146) were significantly younger at the first follow-up measurement (12.63 ± 2.73 years vs. 13.46 ± 3.08 years, *p* = 0.014), but did not differ concerning sex, BMI *z*-score, SCr/*Q*-age, and SCr/*Q*-height. Compared to children with exactly 2 follow-up measurements (*n* = 76), children with ≥ 3 follow-up measurements (*n* = 70) did not differ concerning age, sex, BMI *z*-score, SCr/*Q*-age, and SCr/*Q*-height. Compared to children with exactly 3 follow-up measurements (*n* = 44), children with ≥ 4 follow-up measurements (*n* = 26) were significantly younger at the third follow-up measurement (median 13.94 (IQR 12.68–15.06) years vs. median 16.21 (IQR 14.67–17.43) years, *p* < 0.001), but did not differ concerning sex, BMI *z*-score, SCr/*Q*-age, and SCr/*Q*-height. Compared to children with exactly 4 follow-up measurements (*n* = 16), children with ≥ 5 follow-up measurements (*n* = 10) did not differ concerning age, sex, BMI *z*-score, SCr/*Q*-age, and SCr/*Q*-height during the fourth follow-up measurement.

In Tables 2 and 3, data for paired variables are displayed for females and males, respectively. Inherent to increasing age and the mean baseline age of 12 years of our cohort, weight and height (and thus BSA) increased with each visit in both sexes. BMI *z*-score decreased in both females and males and this reduction was significantly higher in males (*p* < 0.05; with the exception of visits 3 and 4 (*p* = 0.06 and 0.65, respectively)). There was a significant (linear) correlation between BMI *z*-score and fat mass (*r* = 0.59, *p* < 0.0001). SCr increased with each visit with no differences between sexes. As displayed in Tables 2 and 3, rescaled SCr does slightly increase with each visit. Note, in healthy children, SCr/*Q*-age does not change with age, since the normalization with *Q*-age makes SCr/*Q* age- and sex-independent. We applied a linear mixed regression model with random intercept, with SCr/*Q*-age as the dependent variable and age and sex as the independent variables to investigate whether the slope differs from zero or not. The slopes for males (0.01305, *p* = 0.0006) and for females (0.02307, *p* < 0.0001) are significantly different from zero, with an increasing trend in SCr/*Q*-age for both sexes with age. When fat mass was added as an extra covariate (which seriously reduces the number of observations used, therefore not included in Tables 2 and 3), this was not significant. When SCr was rescaled using *Q*-height polynomials, a significant interaction effect (*p* < 0.0001) arises, indicating that SCr/*Q*-height changes differently for males and females (which is unexpected since the SCr-height curve, as defined by Hoste et al. [18], is independent of sex). In this model, the linear increase per visit in SCr/*Q*-height was found to be 0.04820 and 0.003301 in females and males, respectively.

**Table 2:** Follow-up data of females during lifestyle intervention

Visit	Intervention duration (months)	Δ weight (kg)	Δ height (cm)	Δ BSA (m2)	Δ BMI *z*-score	Δ SCr (mg/dL)	Δ SCr/*Q*-age	Δ SCr/*Q*-height
1 (*n* = 158)	14.2 (12.7–16.8)	6.6 (2.6–10.0)	5.6 (1.4–8.5)	0.12 (0.04–0.17)	− 0.04 (− 0.26 to 0.13)	0.04 (0.00–0.09)	0.01 (− 0.06 to 0.09)	0.03 (− 0.06 to 0.09)
2 (*n* = 78)	28.0 (26.0–32.3)	12.5 (6.3–19.2)	11.2 (2.0–15.1)	0.24 (0.10–0.31)	− 0.04 (− 0.42 to 0.24)	0.10 (0.05–0.16)	0.06 (− 0.03 to 0.16)	0.08 (0.01–0.17)
3 (*n* = 34)	41.6 (38.8–47.6)	20.3 (10.4–29.2)	13.5 (2.8–21.5)	0.34 (0.16–0.48)	− 0.14 (− 0.44 to 0.31)	0.18 (0.10–0.23)	0.10 (0.02–0.20)	0.12 (0.05–0.23)
4 (*n* = 11)	60.8 (52.3–69.6)	28.6 (18.5–39.9)	27.7 (21.2–36.6)	0.55 (0.36–0.66)	− 0.33 (− 1.34 to 0.44)	0.23 (0.12–0.26)	0.15 (− 0.01 to 0.24)	0.17 (0.06–0.24)
5 (*n* = 5)	84.5 (60.6–91.1)	57.6 (38.0–61.9)	29.0 (15.4–45.4)	0.89 (0.62–1.00)	0.90 (− 0.18 to 1.10)	0.29 (0.16–0.34)	0.26 (0.01–0.30)	0.23 (0.08–0.30)

Data presented as median (interquartile range). Delta of paired variables was calculated out of value of the last visit minus baseline value

*BSA* body surface area, *BMI* body mass index, *SCr* serum creatinine, *SCr/Q-age* serum creatinine normalized using *Q*-age polynomials, *SCr/Q-height* serum creatinine normalized using *Q*-height polynomials

**Table 3:** Follow-up data of males during lifestyle intervention

Visit	Intervention duration (months)	Δ weight (kg)	Δ height (cm)	Δ BSA (m2)	Δ BMI *z*-score	Δ SCr (mg/dL)	Δ SCr/*Q*-age	Δ SCr/*Q*-height
1 (*n* = 147)	15.4 (13.0–18.6)	8.0 (2.9–13.3)	7.5 (5.2–10.7)	0.13 (0.06–0.24)	− 0.25 (− 0.53 to 0.06)	0.06 (0.01–0.10)	0.00 (− 0.06 to 0.07)	0.00 (− 0.07 to 0.07)
2 (*n* = 68)	29.2 (26.5–34.8)	18.7 (8.8–24.5)	16.1 (11.4–19.7)	0.32 (0.18–0.43)	− 0.29 (− 0.65 to 0.03)	0.12 (0.08–0.19)	0.03 (− 0.06 to 0.11)	0.02 (− 0.05 to 0.09)
3 (*n* = 36)	45.9 (41.1–59.6)	29.8 (19.6–36.7)	21.8 (17.1–28.1)	0.48 (0.34–0.60)	− 0.34 (− 0.65 to 0.08)	0.24 (0.16–0.29)	0.10 (− 0.04 to 0.18)	0.06 (− 0.04 to 0.13)
4 (*n* = 15)	59.8 (54.8–69.5)	37.1 (17.5–42.3)	24.3 (19.7–27.0)	0.56 (0.33–0.65)	− 0.74 (− 0.96 to − 0.15)	0.28 (0.15–0.36)	0.10 (− 0.08 to 0.21)	0.10 (− 0.04 to 0.19)
5 (*n* = 5)	78.2 (69.5–86.7)	35.2 (30.6–50.8)	32.3 (29.6–38.1)	0.63 (0.57–0.83)	− 1.13 (− 1.31 to 0.22)	0.35 (0.14–0.40)	0.09 (− 0.12 to 0.25)	0.19 (− 0.06 to 0.34)

Data presented as median (interquartile range). Delta of paired variables was calculated out of value of the last visit minus baseline value

*BSA* body surface area, *BMI* body mass index, *SCr* serum creatinine, *SCr/Q-age* serum creatinine normalized using *Q*-age polynomials, *SCr/Q-height* serum creatinine normalized using *Q*-height polynomials

### Effect of changes in BMI z-score

As shown in Tables 2 and 3, during the lifestyle intervention BMI *z*-score decreased in both females and males. A linear mixed regression model with random intercept, with BMI *z*-score as the dependent variable and age and sex as the independent variables, demonstrated a significant decrease of BMI *z*-score in males (slope =  − 0.07556, *p* < 0.0001), but not in females (slope =  − 0.01561, *p* = 0.3383).

In order to examine associations between BMI *z*-score reduction and change in SCr/*Q*-age, 70 children with 4 consecutive visits were selected (baseline and 3 visits thereafter), displayed in Tables 4 and 5 for females (*n* = 34) and males (*n* = 36), respectively. Of the females, 61.8%, 41.2%, and 17.6% had a BMI *z*-score reduction of at least 0.00, 0.25, and 0.50, respectively. Of the males, 75.0%, 61.1%, and 36.1% had a BMI *z*-score reduction of at least 0.00, 0.25, and 0.50, respectively. In both sexes, no differences between changes in rescaled SCr could be demonstrated between the children who did reduce in BMI *z*-score and children who did not. We could not demonstrate a significant correlation between the change in BMI *z*-score and change in SCr/*Q*. In the total cohort of children (and not only in the children with 4 consecutive visits), a random intercept linear mixed effects model, using visit, sex, and the interaction of visit with sex, demonstrated a significant increase (positive slope significantly different from zero) in SCr/*Q*-age with visit or SCr/*Q*-height with visit, both in females and males, but more pronounced in females. Even after adjusting for BMI-*z* and age, these effects remained. The same observations were made in the restricted dataset of children with 4 consecutive visits.

**Table 4:** Change in rescaled SCr after 4 consecutive visits in females (*n* = 34), stratified according to BMI *z*-score reduction

	Δ BMI *z* ≥ 0.00 (*n* = 13)	Δ BMI *z* < 0.00 (*n* = 21)	*p*-value	Δ BMI *z* > − 0.25 (*n* = 20)	Δ BMI *z* ≤ − 0.25 (*n* = 14)	*p*-value	Δ BMI *z* > − 0.50 (*n* = 28)	Δ BMI *z* ≤ − 0.50 (*n* = 6)	*P*-value
Δ SCr/*Q*-age	0.12 (0.01–0.18)	0.09 (0.02–0.24)	0.889	0.10 (− 0.02 to 0.19)	0.09 (0.02–0.23)	0.877	0.10 (0.06–0.21)	0.06 (0.00–0.20)	0.581
Δ SCr/*Q*-height	0.18 (0.04–0.26)	0.11 (0.05–0.23)	0.649	0.17 (0.05–0.26)	0.11 (0.05–0.21)	0.569	0.14 (0.05–0.24)	0.10 (0.06–0.22)	0.947

Median intervention duration 41.6 months (interquartile range 38.8–47.6 months). Data presented as median (interquartile range). Delta of paired variables was calculated out of value of the last visit minus baseline value. *P*-value is obtained with the independent samples Mann–Whitney *U* test

*BMI* body mass index, *SCr* serum creatinine, *SCr/Q-age* serum creatinine normalized using *Q*-age polynomials, *SCr/Q-height* serum creatinine normalized using *Q*-height polynomials

**Table 5:** Change in rescaled SCr after four consecutive visits in males (*n* = 36), stratified according to BMI *z*-score reduction

	Δ BMI *z* ≥ 0.00 (*n* = 9)	Δ BMI *z* < 0.00 (*n* = 27)	*P*-value	Δ BMI *z* > − 0.25 (*n* = 14)	Δ BMI *z* ≤ − 0.25 (*n* = 22)	*P*-value	Δ BMI *z* > − 0.50 (*n* = 23)	Δ BMI *z* ≤ − 0.50 (*n* = 13)	*P*-value
Δ SCr/*Q*-age	0.02 (− 0.06 to 0.20)	0.10 (− 0.01 to 0.18)	0.810	0.11 (− 0.06 to 0.19)	0.09 (− 0.02 to 0.19)	0.855	0.10 (− 0.05 to 0.18)	0.08 (0.02–0.20)	0.932
Δ SCr/*Q*-height	0.03 (− 0.04 to 0.14)	0.09 (− 0.05 to 0.13)	0.897	0.03 (− 0.05 to 0.14)	0.10 (− 0.03 to 0.12)	0.803	0.10 (− 0.04 to 0.13)	0.05 (− 0.04 to 0.13)	0.771

Median intervention duration 45.9 months (interquartile range 41.1–59.6 months). Data presented as median (interquartile range). Delta of paired variables was calculated out of value of the last visit minus baseline value. *P*-value is obtained with the independent samples Mann–Whitney *U* test

*BMI* body mass index, *SCr* serum creatinine, *SCr/Q-age* serum creatinine normalized using *Q*-age polynomials, *SCr/Q-height* serum creatinine normalized using *Q*-height polynomials

## Discussion

The main conclusion of this study is that, even though a slight increase in rescaled serum creatinine (SCr/*Q*) is observed, a clinician should not expect a major change in SCr-based estimated kidney function during lifestyle intervention in children with overweight and obesity. During this lifestyle intervention BMI *z*-score decreased in both females and males and this reduction is significantly higher in males. There was no correlation between change in BMI *z*-score and change in SCr/*Q* and no difference in change in SCr/*Q* during follow-up between children who achieved a significant reduction in BMI *z*-score and children who did not.

So how can we interpret the SCr/*Q* increase and how can this minor increase in SCr/*Q* be explained? As described previously [12, 13], the children in this study are not known with overt kidney disease and about 95% of the children in our cohort had a SCr/*Q* in the normal reference range (in other words, a normal estimated kidney function) at baseline. Assuming a normal SCr/Q (a value of “1”) and thus eGFR-FAS of 107.3 mL/min/1.73 m^2^ (since eGFR-FAS equals 107.3/(SCr/*Q*)), the observed about yearly SCr/*Q* increase of 0.01–0.04 equals an eGFR-FAS reduction of about 1.1–4.1 mL/min/1.73 m^2^. Knowing that SCr is a marker for both kidney function and muscle mass, this minor increase in SCr/*Q* over time cannot be interpreted easily and we come to the following hypotheses.

First, the fact that SCr itself increases with each visit during this lifestyle intervention program is not abnormal in children aged around 12 years. Namely, each visit goes hand in hand with age, and increasing age means increasing muscle mass, hence, increasing SCr [11]. Although, since SCr/*Q* is independent of sex and age (*Q*-age), or height (*Q*-height), one should not expect that an increase in SCr/*Q* can be explained by merely increasing age.

Second, it seems plausible that during a lifestyle intervention program, the amount of muscle mass not only increases with age but also results from more physical activity. Increase in muscle mass leads to higher SCr levels and, since this effect is age and height independent, also increased SCr/*Q*. In this study, we determined the percentage of fat mass using air displacement plethysmography in a randomly selected group of children. When fat mass was added as an extra covariate (which seriously reduces the number of observations used which is a limitation of this study), a significant effect on change in SCr/*Q* could not be demonstrated.

Finally, there is the hyperfiltration hypothesis. Multiple studies demonstrate that obesity-related hyperfiltration might be reversible in case of weight loss [27, 28]. So, one should expect that during lifestyle intervention, this is also the case in children in whom obesity-related hyperfiltration might already be present. As a result, it can be expected that this supraphysiological GFR decreases, which corresponds with increase in SCr or SCr/*Q*. In the present study however, we could not demonstrate a correlation between change in BMI *z*-score and change in SCr/*Q*, nor a difference in change in SCr/*Q* during follow-up between children who achieved a significant reduction in BMI *z*-score and children who did not.

In this study, both *Q*-age [19] and *Q*-height [18] were used to rescale SCr and to correct for sex and age, and height, respectively. The fact that a difference in SCr/*Q* evolution between males and females is demonstrated when *Q*-height is used as the scaling factor (something that is not seen when *Q*-age is used to scale SCr) is intriguing and deserves further investigation. Note that the SCr-height curve, as defined by Hoste et al. [18], is independent of sex.

To the best of our knowledge, the present study is the first to evaluate the course of SCr levels during a lifestyle intervention in children with overweight and obesity. However, this study is not without limitations. First, this study did not include measured GFR. GFR was estimated with SCr, though other endogenic biomarkers, such as cystatin C, could be of interest as well. Second, loss to follow-up was highly present. While this is inherent in a lifestyle intervention program, a dropout of about 50% of children about each year leads to bias. Third, this study lacks a control group. We believe, though, this is only a minor limitation, since our cohort size justified stratification according to BMI *z*-score reduction or not, and the course of SCr in healthy children without lifestyle intervention is already known. Finally, though we included gold standard measurement of body proportions using air displacement plethysmography, due to missing data, group size significantly dropped when fat mass was added as an independent variable. As body composition is a well-known confounder of SCr, it is a serious limitation of this study that fat and fat-free masses were only measured in a minority of the children. Because of this, in line with other studies [16], effect of the lifestyle intervention was defined as BMI *z*-score reduction, while BMI *z*-score has serious limitations (e.g., it does not discriminate weight gain resulting from excess muscle mass or fat mass, nor does it discriminate between central and visceral adiposity).

In conclusion, this study demonstrates that rescaled serum creatinine (SCr/*Q*) slightly increases during lifestyle intervention in children with overweight and obesity. This effect seems to be independent from change in BMI *z*-score. Whether this minor change in estimated kidney function has clinical consequences in the long-term remains to be seen in trials with a longer follow-up period.

## Supplementary Information

Below is the link to the electronic supplementary material.

Graphical abstract (PPTX 88 KB)

## Data Availability

The datasets generated during and/or analyzed during the current study are available from the corresponding author on reasonable request.
