# Supplementary material for: The evaluation of kidney function estimation during lifestyle intervention in children with overweight and obesity
Source: Pediatr Nephrol. 2024 Jul 4;39(11):3271–8. doi: 10.1007/s00467-024-06435-0 (PMC11413135; doi:10.1007/s00467-024-06435-0)
Supplement: Supplementary file 1 — Graphical abstract (PPTX 88 KB) [file 467_2024_6435_MOESM1_ESM.pptx]

## Slide 1
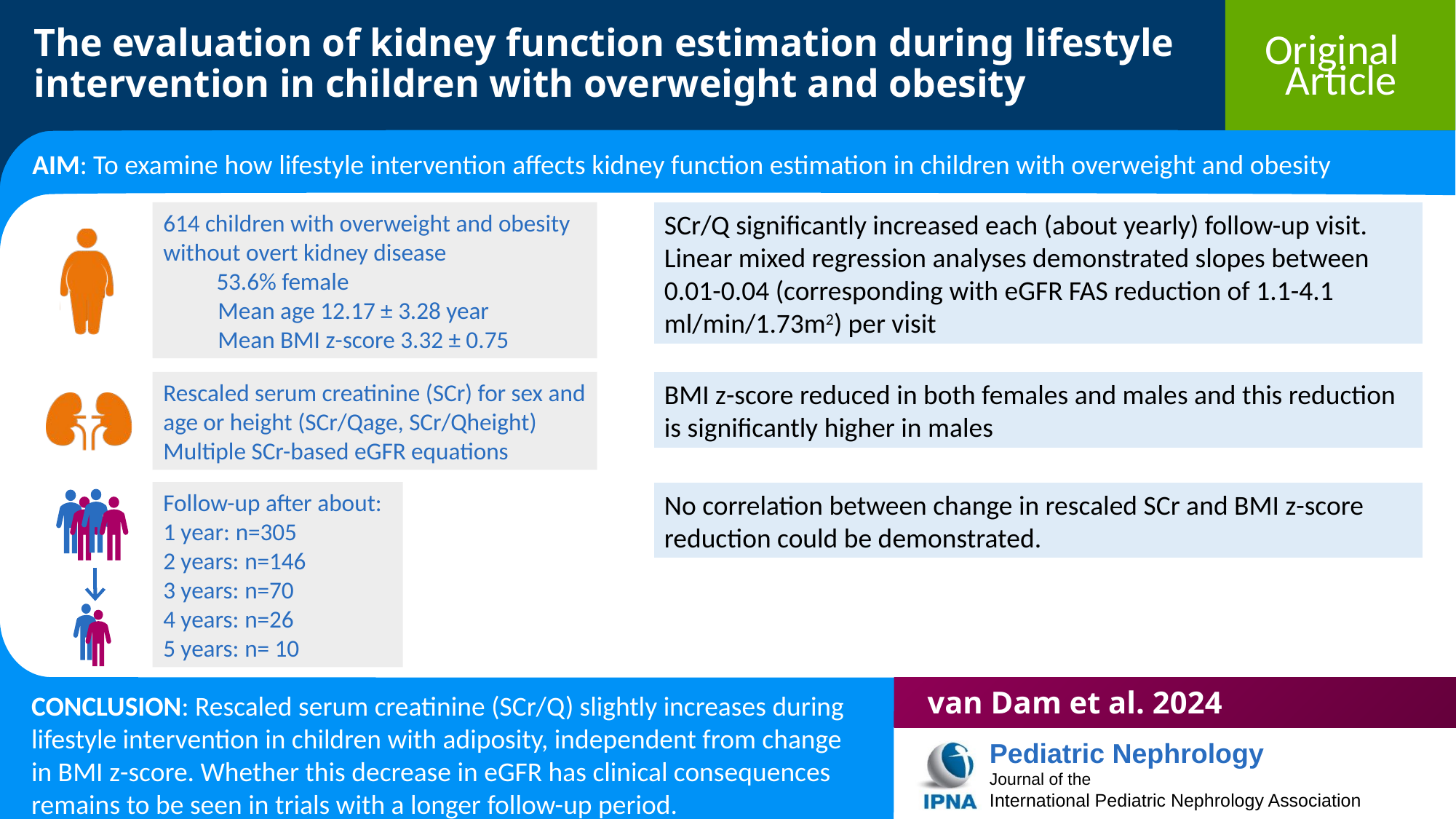

The evaluation of kidney function estimation during lifestyle intervention in children with overweight and obesity
AIM: To examine how lifestyle intervention affects kidney function estimation in children with overweight and obesity
SCr/Q significantly increased each (about yearly) follow-up visit. Linear mixed regression analyses demonstrated slopes between 0.01-0.04 (corresponding with eGFR FAS reduction of 1.1-4.1 ml/min/1.73m2) per visit
614 children with overweight and obesity without overt kidney disease
53.6% female
Mean age 12.17 ± 3.28 year
Mean BMI z-score 3.32 ± 0.75
Rescaled serum creatinine (SCr) for sex and age or height (SCr/Qage, SCr/Qheight)
Multiple SCr-based eGFR equations
BMI z-score reduced in both females and males and this reduction is significantly higher in males
No correlation between change in rescaled SCr and BMI z-score reduction could be demonstrated.
Follow-up after about:
1 year: n=305
2 years: n=146
3 years: n=70
4 years: n=26
5 years: n= 10
van Dam et al. 2024
CONCLUSION: Rescaled serum creatinine (SCr/Q) slightly increases during lifestyle intervention in children with adiposity, independent from change in BMI z-score. Whether this decrease in eGFR has clinical consequences remains to be seen in trials with a longer follow-up period.
